# Supplementary material for: Deficits in decision-making induced by parietal cortex inactivation are compensated at two time scales
Source: Neuron. Author manuscript; Available in PMC 2023 Jun 15. (PMC9233071; doi:10.1016/j.neuron.2022.03.022)
Supplement: 2 [file NIHMS1791980-supplement-2.pdf]

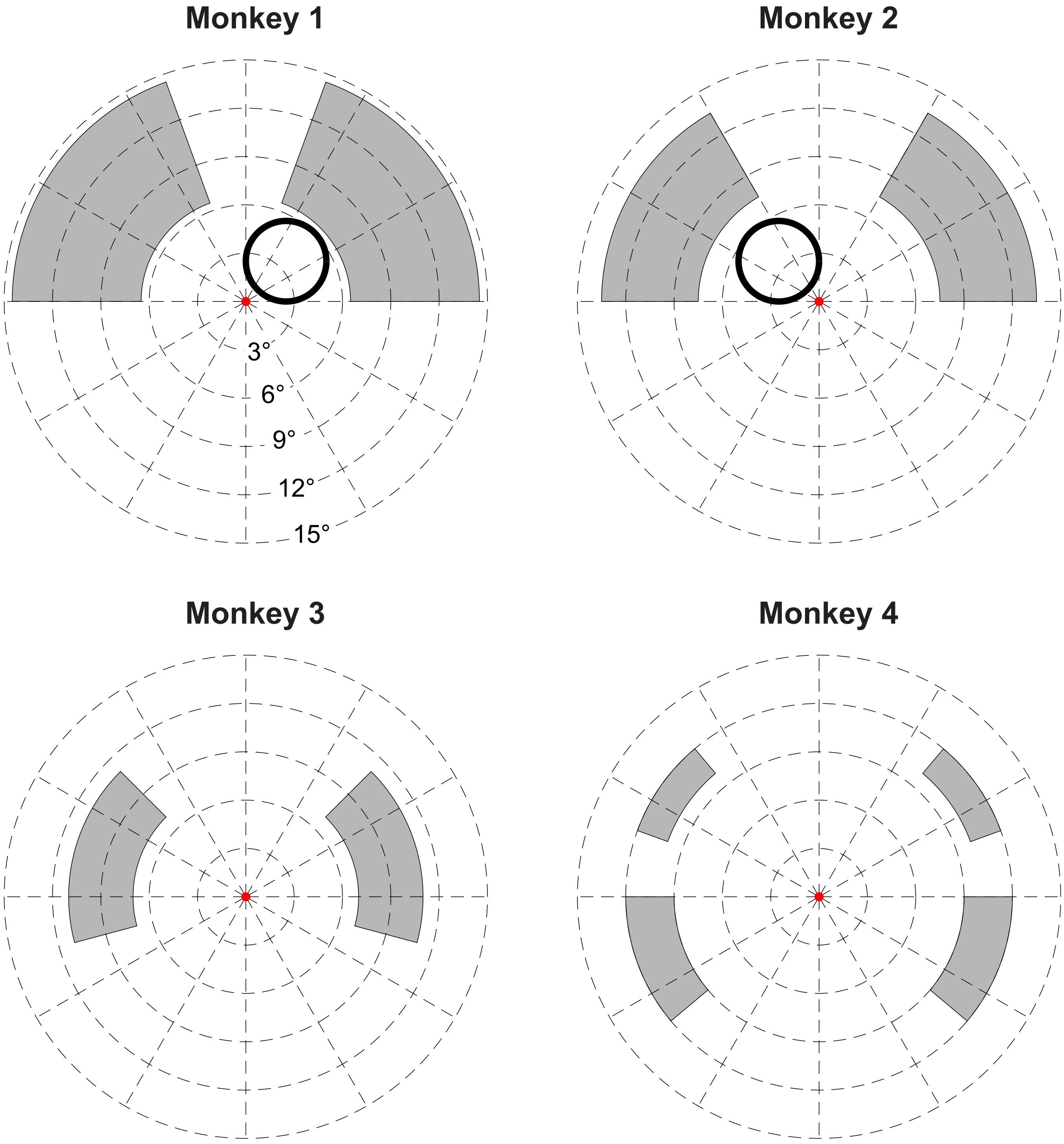

Figure S1. Stimulus configuration (Related to Figure 1).The left and right choice-targets were positioned randomly on each trial using independent samples from the shaded regions of the visual field (uniform distribution over range of *r*,*θ*). For monkey 4, both choice targets were in either the upper or the lower hemifield. The area subtended by the random dot motion display (black circles; Monkeys 1 and 2) was consistent across trials/sessions and confined to the hemifield ipsilateral to the inactivated cortex. Eccentricities are in degrees visual angle.

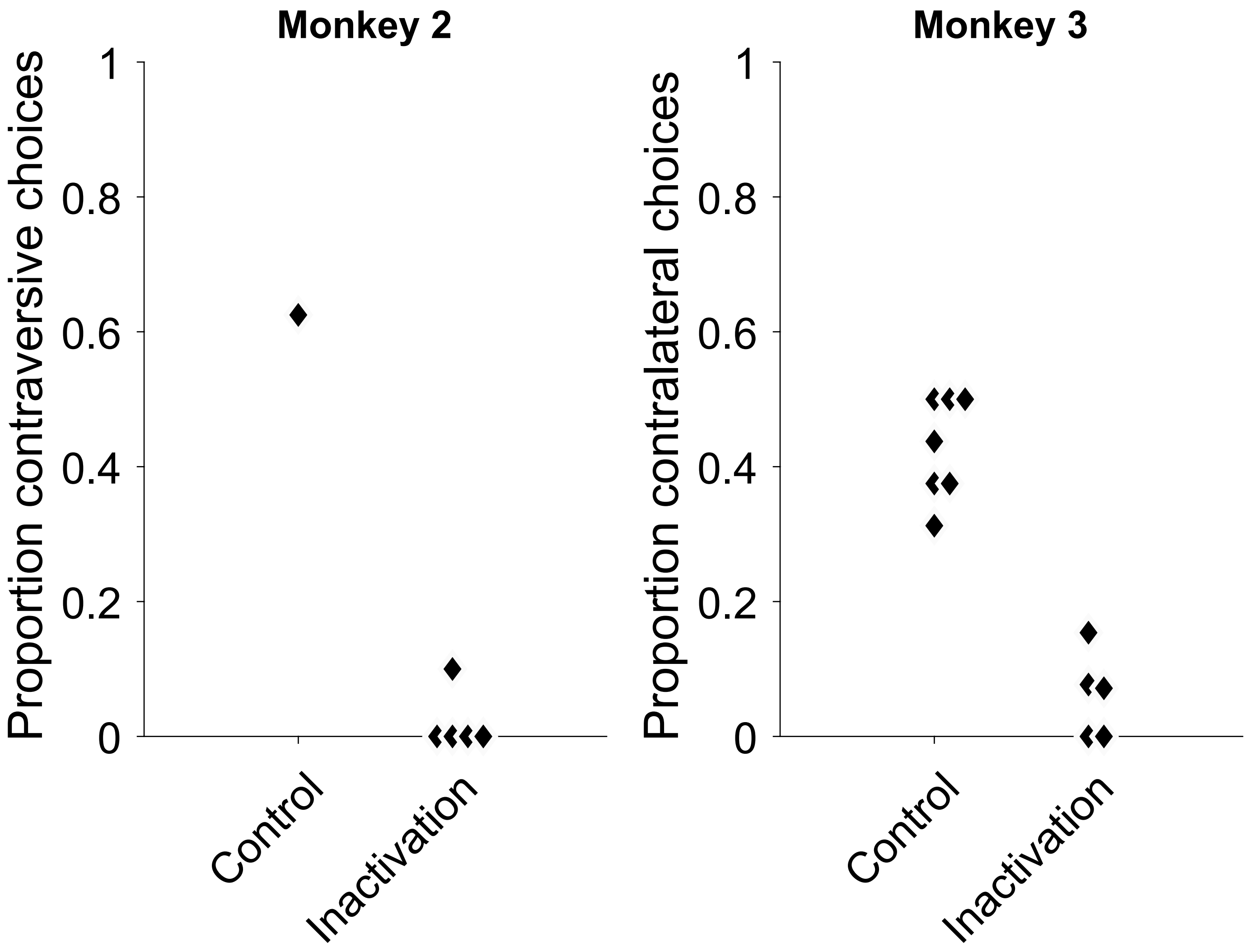

Figure S2. Behavior on the side-preference task (Related to Figure 2). After completion of the main experiment, Monkeys 2 and 3 were tested on a preference task intended to approximate a neurological double-simultaneous stimulation test for extinction.The experimenter presented desirable food items to the monkey's left and right side, equidistant from its mouth. The monkey indicated its preferred item by picking it with its tongue. The proportion of chosen items from the side contralateral to the inactivated cortex is shown. Points are data from one session. Points belonging to the same treatment group (control or muscimol inactivation) are displaced horizontally for visualization.

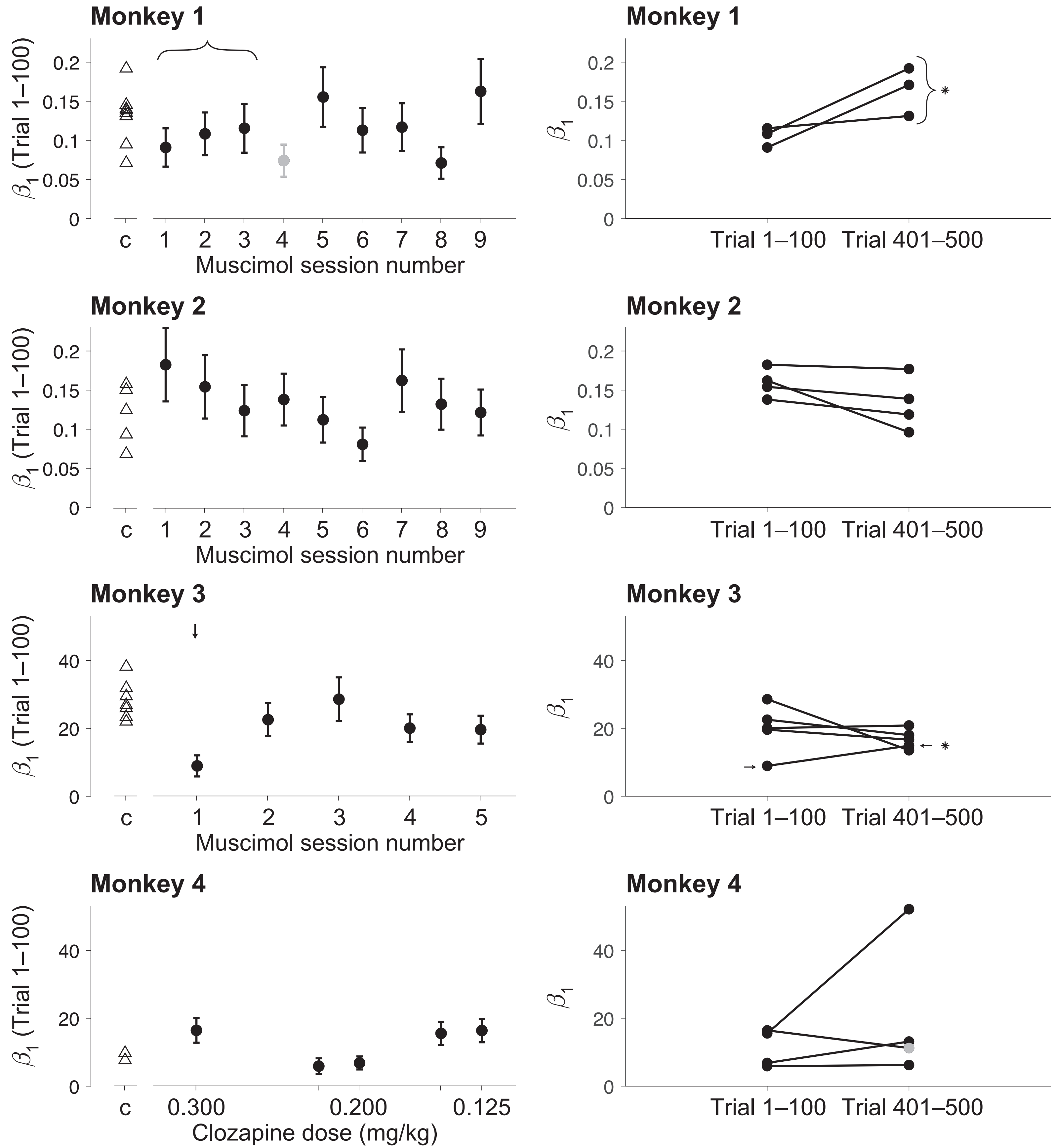

Figure S3. Effect of inactivation on sensitivity (Related to Figure 4). Data are from the same sessions as in Figure 4A–D. Here we show the values of the sensitivity parameter β_1. *Left column* shows sensitivity in the first 100 trials of each of the sessions. Inactivation led to reduced sensitivity in Monkey 1 (combined data from the first three sessions; curly brace) and in Monkey 3 (first session; arrow). *Right column* shows the comparison of sensitivity (β_1) in the first 100 trials with trials 401-500 in the same session. All experiments with significant **bias** effect are shown (i.e., same experiments as in Figure 4C–D). These are the same experiments that exhibit reliable changes in sensitivity (Monkey 1) and they include the 1 experiment in Monkey 3 that revealed a change in sensitivity. Asterisks identify significant change in sensitivity between early and late trials, consistent with compensation. This applies to the grouped data for Monkey 1 and the first session for Monkey 3. Many sessions with significant changes in bias were not accompanied by reliable changes in sensitivity.

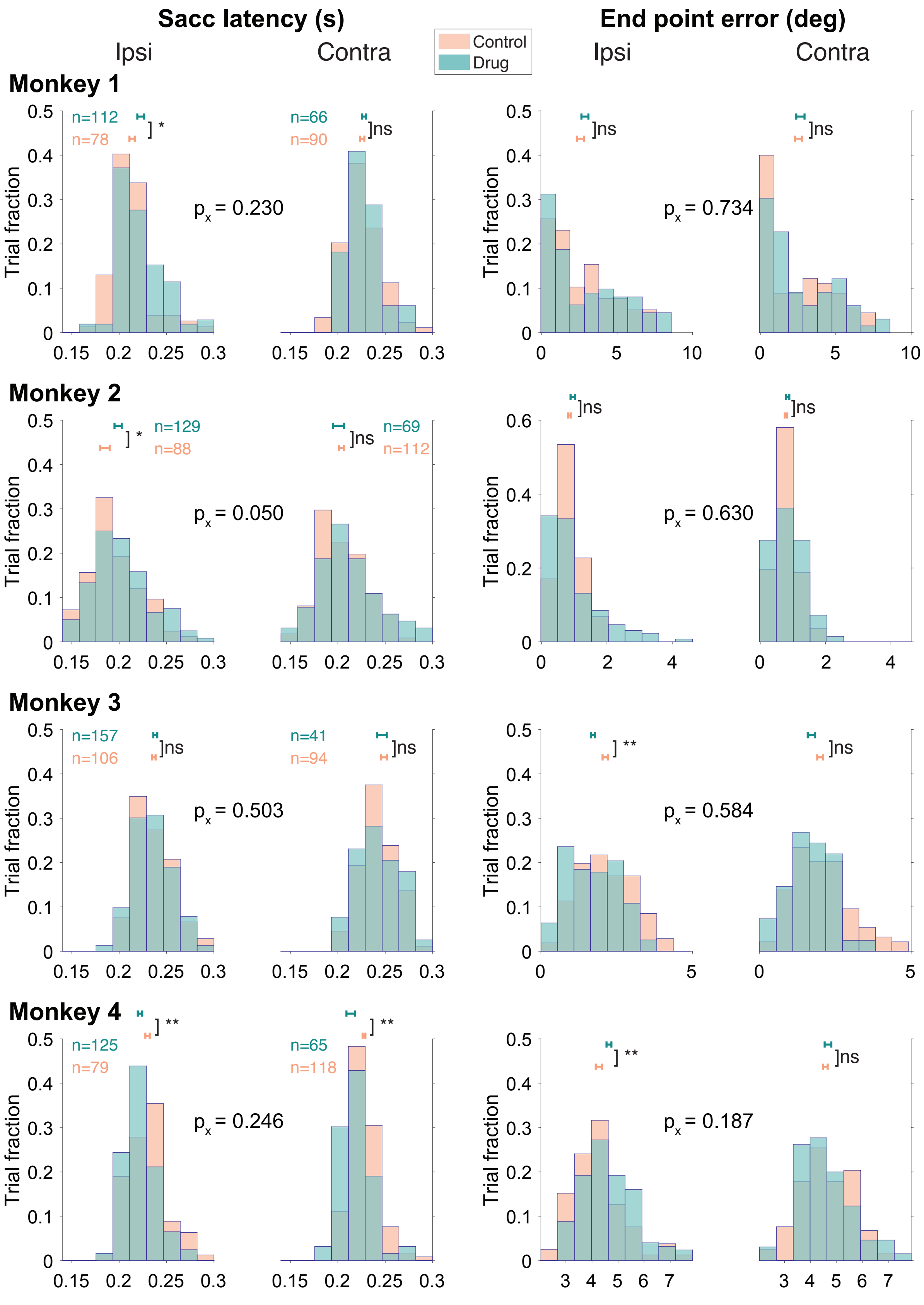

Figure S4. Analysis of saccade metrics for the four monkeys (Related to STAR Methods). Distributions of saccadic latency (left) and end-point error (right) are shown separately for the four monkeys (rows). Colors indicate inactivation (cyan) and control (pink) sessions. Each plot contains a pair of overlaid histograms for ipsiversive and contraversive saccades. The small horizontal error bars are ± 1 s.e.m. centered at the means. Asterisks indicate significant differences in the means for inactivation versus control (*, **: $p < 0.05$, $p < 0.01$; ns: not significant). P-values specified between the left and right histograms (p_x) refer to the interaction between side and drug effects (Equation 10). An effect of inactivation on these saccade metrics was not detectable. Saccadic peak velocities (not shown) were similarly unaffected.

	pre vs. post			control vs post		
	value	SE	p	value	SE	p
Monkey 1	-0.77	0.34	0.02	-0.98	0.25	9e-05
Monkey 2	-1.08	0.40	0.01	-1.58	0.32	1e-06
Monkey 3	-2.31	0.50	4e-06	-1.90	0.42	6e-06
Monkey 4	-1.66	0.37	6e-06	-2.20	0.34	1e-10

Table S1. β2 values, SE, and p values from Equation 5 (Related to STAR Methods and Figure 3).

	pre vs. post			control vs post		
	value	SE	p	value	SE	p
Monkey 1	-0.02	0.03	0.58	-0.03	0.03	0.32
Monkey 2	0.03	0.06	0.63	0.07	0.05	0.13
Monkey 3	-16.68	6.61	0.01	-17.77	3.87	4e-06
Monkey 4	-1.38	3.11	0.66	-2.79	2.78	0.32

Table S2. β3 values, SE, and p values from Equation 5 (Related to STAR Methods and Figure 3).

Monkey	Task	Inactivation Method	Date	Session Type	Electrode Depth (µm)	Pipette Depth (µm)	Distance electrode to pipette (µm)	Injection Speed (µL/min)	Injected Volume	Drug Dose	Additional Info
1	Motion	Pharma-cology	20180109	Sham	-	-	-	-	-	-	
			20180121	Muscimol	8000	8500, 6700, 4900, 3100	x=1000, y=2000, z=500, D=2291	0.3	15 + 10 + 12 + 8 = 45µL	8µg/µL	1
			20180626	Muscimol	6500	8000, 6200, 4400, 2600	x=1000, y=2000, z=1500, D=2693	0.3	3 + 6 + 6 + 4 = 19µL	8µg/µL	
			20180701	Muscimol	6500	8000, 6200, 4400, 2600	x=1000, y=2000, z=1500, D=2693	0.3	4 + 6 + 6 + 4 = 20µL	8µg/µL	%
			20180705	Saline	6500	8000, 6200, 4400, 2600	x=1000, y=2000, z=1500, D=2693	0.3	4 + 6 + 6 + 4 = 20µL	-	%
			20180803	Saline	7100	8000, 6200, 4400, 2600	x=1000, y=2000, z=900, D=2410	0.3	1 + 1 + 1 + 1 = 4µL	-	
			20180807	Muscimol	6500	8000, 6200, 4400, 2600	x=1000, y=2000, z=1500, D=2693	0.3	2 + 2 + 2 + 2 = 8µL	8µg/µL	#
			20180814	Sham	-	-	-	-	-	-	
			20180815	Saline	6500	8000, 6200, 4400, 2600	x=1000, y=2000, z=1500, D=2693	0.3	4 + 6 + 6 + 4 = 20µL	-	
			20180816	Muscimol	6500	8000, 6200, 4400, 2600	x=1000, y=2000, z=1500, D=2693	0.3	4 + 6 + 6 + 4 = 20µL	8µg/µL	
			20180821	Sham	6900	-	-	-	-	-	*
			20180822	Saline	6500	8000, 6200, 4400, 2600	x=1000, y=2000, z=1500, D=2693	0.3	4 + 6 + 6 + 4 = 20µL	-	
			20180823	Saline	6500	8000, 6200, 4400, 2600	x=1000, y=2000, z=1500, D=2693	0.3	4 + 6 + 6 + 4 = 20µL	-	
			20180824	Muscimol	6500	8000, 6200, 4400, 2600	x=1000, y=2000, z=1500, D=2693	0.3	4 + 6 + 6 + 4 = 20µL	8µg/µL	*
			20180829	Saline	6500	8000, 6200, 4400, 2600	x=1000, y=2000, z=1500, D=2693	0.3	4 + 6 + 6 + 4 + 20µL	-	
			20180830	Muscimol	7150	8000, 6200, 4400, 2600	x=1000, y=2000, z=850, D=2392	0.3	4 + 6 + 6 + 4 = 20µL	8µg/µL	
			20180904	Muscimol	6500	8000, 6200, 4400, 2600	x=1000, y=2000, z=1500, D=2693	0.3	4 + 6 + 5.5 + 4.5 = 20µL	8µg/µL	
			20180914	Muscimol	6500	8000, 6200, 4400, 2600	x=1000, y=2000, z=1500, D=2693	0.3	4 + 6 + 6 + 4 = 20µL	8µg/µL	
2	Motion	Pharma-cology	20190808	Muscimol	3650	8000, 6200, 4400, 2600	x=2000, y=1000, z=4350, D=4891	0.4	4 + 6 + 6 + 4 = 20µL	8µg/µL	1
			20190814	Muscimol	3500	8000, 6200, 4400, 2600	x=2000, y=1000, z=4500, D=5025	0.4	4 + 6 + 6 + 4 = 20µL	8µg/µL	
			20190829	Saline	3600	8000, 6200, 4400, 2600	x=2000, y=1000, z=4400, D=4936	0.4	5 + 5 + 6 + 4 = 20µL	-	
			20190902	Muscimol	3500	8000, 6200, 4400, 2600	x=2000, y=1000, z=4500, D=5025	0.4	4 + 6 + 6 + 4 = 20µL	8µg/µL	N
			20190913	Sham	3600	-	-	-	-	-	
			20191002	Sham	3600	-	-	-	-	-	
			20191003	Muscimol	3600	8000, 6200, 4400, 2600	x=3000, y=1000, z=4400, D=5418	0.4	4 + 6 + 6 + 4 = 20µL	8µg/µL	N
			20191008	Sham	7000	-		-	-	-	
			20191009	Muscimol	6800	8000, 6200, 4400, 2600	x=4000, y=1000, z=1200, D=4294	0.4	4 + 6 + 6 + 4 = 20µL	8µg/µL	
			20191015	Muscimol	6600	8000, 6200, 4400, 2600	x=3000, y=1000, z=1400, D=3458	0.4	4 + 6 + 6 + 4 = 20µL	8µg/µL	
			20191029	Sham	6150	-		-	-	-	N
			20191030	Muscimol	5400	8000, 6200, 4400, 2600	x=4000, y=1000, z=2600, D=4874	0.4	4 + 6 + 6 + 4 = 20µL	8µg/µL	N
			20191106	Muscimol	6500	8000, 6200, 4400, 2600	x=4000, y=1000, z=1500, D=4387	0.4	4 + 6 + 6 + 4 = 20µL	8µg/µL	N
			20191111	Muscimol	1700	8000, 6200, 4400, 2600	x=2000, y=1000, z=6300, D=6685	0.4	4 + 6 + 6 + 4 = 20µL	8µg/µL	N
3	Time	Pharma-cology	20200121	Sham	4000	-	-	-	-	-	N
			20200122	Sham	4000	-	-	-	-	-	N
			20200128	Sham	4100	-	-	-	-	-	N
			20200207	Muscimol	8000	8500, 6700, 4900, 3100	x=2000, y=0, z=500, D=2062	0.4	4 + 6 + 6 + 4 = 20µL	8µg/µL	N, 1
			20200213	Muscimol	6850	8500, 6700, 4900, 3100	x=2000, y=0, z=1650, D=2593	0.4	4 + 6 + 6 + 4 = 20µL	8µg/µL	N
			20200218	Sham	6500	-	-	-	-	-	N
			20200221	Sham	4800	-	-	-	-	-	N
			20200225	Muscimol	5200	8500, 6700, 4900, 3100	x=3000, y=0, z=3300, D=4460	0.4	4 + 6 + 6 + 4 = 20µL	8µg/µL	N
			20200303	Sham	3000	-	-	-	-	-	N
			20200304	Muscimol	4500	8500, 6700, 4900, 3100	x=4000, y=1000, z=4000, D=5745	0.4	4 + 6 + 6 + 4 = 20µL	8µg/µL	N
			20200310	Sham	4100	-	-	-	-	-	N
			20200311	Muscimol	4500	8500, 6700, 4900, 3100	x=4000, y=1000, z=4000, D=5745	0.4	4 + 6 + 6 + 4 = 20µL	8µg/µL	!, N
4	Time	Chemo-genetics	20171121	Viral injection	13 locations, each spaced 500µm apart, between 9800 and 3800	13 locations, each spaced 500µm apart, between 9000 and 3000	800	0.1	13 injections of 0.5µL each. Total volume: 6.5 µL	-	vi
			20171122	Viral injection	14 locations, each spaced 500µm apart, between 9700 and 3200	14 locations, each spaced 500µm apart, between 9000 and 2500	700	0.1	14 injections of 0.5µL each. Total volume: 7µL	-	vi
			20180522	Clozapine	9000	-	-	-	-	0.125 mg/kg	v
			20180604	Clozapine	7000	-	-	-	-	0.300 mg/kg	v, *, 1
			20180608	Clozapine	-	-	-	-	-	0.150 mg/kg	
			20180727	Saline	-	-	-	-	-	-	
			20180731	Clozapine	-	-	-	-	-	0.200 mg/kg	
			20180807	Saline	-	-	-	-	-	-	
			20190809	Clozapine	-	-	-	-	-	0.225 mg/kg	

Table S3. List of all experimental sessions (Related to STAR Methods). Sessions are sorted by date for each monkey. The electrode and pipette depths are in micrometers below the dura. Electrode depth was constant throughout the session and we list the depth of either the tip of the electrode (single channel) or deepest electrode (24-channel V-probe). For muscimol infusion, the pipette was placed at four different depths. The injected volume is reported for each depth. For the muscimol infusion experiments, the distance between the recording electrode and the deepest pipette location is reported (i.e., first injection site).

Symbols and abbreviations:
Motion: Motion direction task.
Time: Temporal order task.
*: Monkey completed < 500 trials post injection.
%: Strongest motion strength (coherence +/-51.2%) was not used in this session.
#: Low-volume muscimol injection session.
!: Last session in Monkey 3. Further sessions were not possible due to the health and safety restrictions related to the COVID-19 pandemic.
vi: Viral vector injection session.
v: Sessions with V-probe recordings. Data shown in Figure 2D.
N: Collected data on side-preference task.
1: First (high-dose) session. Data shown in Figure 3.
